# Supplementary material for: Unprecedented Fine Structure of a Solar Flare Revealed by the 1.6 m New Solar Telescope
Source: Sci Rep. 2016 Apr 13;6:24319. doi: 10.1038/srep24319 (PMC4829858; doi:10.1038/srep24319)
Supplement: Supplementary movie Legend [file srep24319-s1.pdf]

Unprecedented Fine Structure of a Solar Flare Revealed by the 1.6 m New Solar Telescope

Ju Jing*[1,2], Yan Xu[1,2], Wenda Cao[1,2], Chang Liu[1,2], Dale Gary[1,2], and Haimin Wang[1,2]

[1] *Center For Solar-Terrestrial Research, New Jersey Institute of Technology, University Heights, Newark, NJ 07102-1982, USA*

[2] *Big Bear Solar Observatory, New Jersey Institute of Technology, 40386 North Shore Lane, Big Bear City, CA 92314-9672, USA*

March 14, 2016

Supplementary Video Legends

Supplementary Video 1 | BBSO NST/VIS Hα+1.0Å image sequence.

The Hα+1.0Å images are displayed at one third of the original spatial resolution. In the plot of the GOES soft X-ray 1-8 Å light curve, the time of each Hα+1.0Å image is indicated with the vertical line.

*Correspondence and requests for materials should be addressed to J.J. (email: ju.jing@njit.edu)
